# Effects of maize straw and corncob return on the soil quality and on the soil microbial structures and functions

**DOI:** 10.3389/fmicb.2025.1675172

**Published:** 2025-11-12

**Authors:** Yu Zhong, Ruoyu Li, Jiayi E, Hai Chi, Ning Cao, Zhongyi Bai, Xinglin Du, Le Wang

**Affiliations:** 1 Jilin Provincial Engineering Laboratory of Plant Genetic Improvement, College of Plant Science, Jilin University, Changchun, China; 2 Key Laboratory of Inland Saline-Alkaline Aquaculture, Ministry of Agriculture and Rural Affairs, East China Sea Fisheries Research Institute, Chinese Academy of Fishery Sciences, Shanghai, China

**Keywords:** straw return, metagenomic, maize, soil quality, microbial structure

## Abstract

Straw return is an effective agricultural strategy for incorporating organic carbon into soil organic matter pools through microbial decomposition. This process modifies soil physicochemical properties, thereby altering microbial habitats and resource availability, which can influence the structure and function of soil microbial communities. However, the changes of soil physicochemical properties and microbial communities under different straw incorporation forms remain poorly understood. And how these straw return materials alter soil physicochemical properties and microbial communities within a single cycle. In this study, we conducted straw returning experiments in a maize-producing region of Jilin Province, China, comparing the impact of two distinct maize-derived residues (crushed maize straw and crushed corncob) on soil quality and microbial communities. Our results demonstrated that corncob return more effectively improved key soil physicochemical properties compared to maize straw return. While neither residue significantly alters microbial alpha diversity, both induced shifts in beta diversity. We identified distinct correlations between dominant microbial taxa and key soil physicochemical parameters. Furthermore, KEGG and GO analyses revealed that both of the residues altered microbial functional hierarchies, with corncob return inducing more pronounced changes than maize straw return. These findings provide a mechanistic basis for optimizing straw management strategies to enhance microbial-mediated soil fertility.

## 1 Introduction

Straw, a carbon-enriched agricultural waste, is rich in nitrogen, phosphorous, potassium, and various micronutrients essential for crop growth (Jin et al., 2020). It plays a pivotal role in replenishing organic carbon (C), nitrogen (N), and phosphorus (P), thereby mitigating soil nutrient imbalances. China produces annual straw of production over 1 billion tons, yet a significant portion is discarded without effective utilization, leading to a myriad of environmental and social challenges. Straw return, recognized as an efficient method of straw utilization, not only maximizes the use of agricultural residues but also protects the environment, earning strong advocacy from both government and scientific communities (Liu et al., 2014; Cai et al., 2018).

Straw return effectively enhances soil aggregate structure and improves soil properties (Jin et al., 2020; Hu et al., 2021), addressing critical issues in agricultural development such as soil degradation, loss of soil organic carbon (SOC) and nutrients, and declining soil fertility. Healthy soil is vital for crop growth and, by extension, human health (Yu et al., 2019). However, the traditional approach of directly returning crop straw to the field presents numerous challenges in terms of straw resource utilization. For instance, crop straw is bulky, and practices such as straw mulching and shallow plowing are commonly used, often resulting in slow straw decomposition, hindered absorption and utilization of straw nutrients, limited improvement in soil organic matter, and reduced crop yields (Dong et al., 2018; Hu et al., 2021).

Soil microbes play an important role in the transformation of straw organic carbon into soil organic carbon, especially in the process of straw decomposition (Marschner et al., 2011). For example, in the first stage, bacteria (e.g., phyla Proteobacteria, Actinobacteria, and Acid-obacteria) dominate microbial communities and mainly mediate the degradation of easily decomposed organic matter such as sugar and fat in straw, however, fungi (e.g., phyla Ascomycota and Basidiomycota) dominate the latter stage and mainly degrade lignin, cellulose and other refractory substances (Marschner et al., 2011). In this process, soil microbes affect C cycling by influencing soil C of different fractions (Six et al., 2006).

At the same time, straw incorporation directly affects the community structure of soil microorganism, because the incorporation alters their habitat and provides abundant carbon sources (Wang et al., 2014). Previous studies showed that soil microbial communities were affected by the placement of straw in the field, straw forms and straw types. Koullas et al. (1992) found that the smaller the straw form, the faster its degradation by microorganisms, changing the composition of bacterial and fungal populations. It can be seen that different straw incorporation forms inevitably lead to dramatic changes in soil microorganisms. Studying the relationship between this change and soil organic carbon fractions will help us to understand the microbial mechanism of soil organic C pool improvement. Though, many scholars have conducted extensive research on straw return mode, deep mechanism analysis still remains scarce.

Jilin province, a vital maize producing region in Northeast China, generates substantial maize residues. Therefore, we carried out straw placement experiments in the maize growing area of Jilin province and compared the effects of two different maize residues (crushed maize straw and crushed corncob) on the soil within a single cycle (from the maize harvest until the sowing of the next season). The objectives were to: (i) evaluate the effects of different forms of straw return on soil characteristics and the changes in microbial community structure; (ii) illustrate the interactions between soil microbial communities and C fractions; (iii) identify an appropriate straw incorporation management in the locality. We hypothesized that different forms of straw would have apparent effects on SOC concentrations. Moreover, straw return would improve soil microbial diversity and C-related microbial abundance in subsoils. This study will provide a new perspective for studying the biological mechanism of organic C accumulation in straw management.

## 2 Materials and methods

### 2.1 Experimental design and sample collection

The field experiment was carried out from October 2023 to April 2024 at the maize fields located in Jilin University farm, Jilin Province (45°3′5.64840″N, 123°13′19.801200″E). Two kinds of straws used in this study were crushed maize straw and crushed corncob. There were three treatments: no straw returned (S); crushed maize straw return (MS); crushed corncob return (CC). After maize harvest in October 2023, the maize field was treated according to the treatment design. And the soil samples were collected in April 2024. We also collected the soil sample from the maize field without straw return in October 2023 and used for the experimental control (CK). Three replicated plots were set up for each treatment (2 m × 2 m for each plot). The previous study reported that maize straw contained 416.2 g/kg total carbon (Pei et al., 2015). In corncob, it contained 411.6 g/kg total carbon (Pahla et al., 2017). Therefore, an equivalent mass of straw materials was applied to all treatments.

For each replicate, five samples were obtained from designated plots within a 2 m × 2 m area in the experimental field. These samples were collected from the corners and center of the designated plot, in accordance with the sampling protocol established by Guo et al. (2022). To minimize disturbance to the soil, soil was carefully extracted using a gardening fork and shovel, soil samples were placed in sterile plastic bags. These samples were subsequently transported to the laboratory in an icebox to maintain optimal conditions. In the laboratory, soil tightly adhering to the maize straws was carefully brushed off. The soil from five individual samples within one plot, serving as a replicate, was pooled and thoroughly mixed to ensure uniformity. One portion of this soil was immediately stored at −80 °C for DNA extraction and sequencing. The remaining sample was utilized for the measurement of various soil parameters, including pH, salinity, soil organic carbon (SOC), total nitrogen (TN), soil alkaline hydrolysis nitrogen (AN), available phosphorus (AP), and available potassium (AK).

### 2.2 Measurement of soil physical and chemical properties

Soil pH was investigated using a pH meter with a soil-water ratio of 1:2.5. Salinity was measured using a salinity meter with a soil-water ratio of 1:5. Soil organic carbon (SOC) content was investigated using potassium dichromate oxidation (Mebius, 1960). Total nitrogen (TN) content was determined using the Kjeldahl method (Bremner, 1960). Soil alkaline hydrolysis nitrogen (AN), available phosphorus (AP) and available potassium (AK) were measured according to Soil Agrochemical Analysis Method (Page, 1982).

### 2.3 DNA extraction, library construction, and metagenomic sequencing

Total genomic DNA was extracted from soil samples using the Mag-Bind^®^ Soil DNA Kit (Omega Bio-tek, Norcross, GA, USA) according to manufacturer’s instructions. The concentration and purity of extracted DNA was determined with TBS-380 and NanoDrop2000, respectively. The quality of the DNA extract was checked on a 1% agarose gel. The DNA extract was fragmented to an average size of about 400 bp using the Covaris M220 (Gene Company Limited, China) for paired-end library construction. A paired-end library was constructed using NEXTFLEX Rapid DNA-Seq (Bioo Scientific, Austin, TX, USA). Adapters containing the full complement of sequencing primer hybridization sites were ligated to the blunt-end of fragments. Paired-end sequencing was performed on an Illumina NovaSeq (Illumina Inc., San Diego, CA, USA) using NovaSeq 6000 S4 Reagent Kit v1.5 following the manufacturer’s instructions.^1^

### 2.4 Sequence quality control and genome assembly

The raw reads were trimmed of adaptors, and low-quality reads (length < 50 bp or with a quality value < 20 or having N bases) were removed by fastp v0.20.0 (Chen et al., 2018). Clean reads after the quality control were assembled using MEGAHIT v1.1.2 (Li et al., 2015). Contigs with a length ≥ 300 bp were chosen as the final assembling result and used for following gene prediction and annotation.

### 2.5 Gene prediction, taxonomy, and functional annotation

Open reading frames (ORFs) from each assembled contigs were predicted using Prodigal. The predicted ORFs with a length ≥ 100 bp were retrieved and translated into amino acid sequences using the NCBI translation table.^2^ A non-redundant gene catalog was constructed using CD-HIT v4.6.1 (Fu et al., 2012) with 90% sequence identity and 90% coverage. Gene abundance of non-redundant genes was estimated for each sample by SOAPaligner v2.21 with 95% identity (Gu et al., 2013).

The non-redundant gene catalog was aligned against the NCBI NR database using DIAMOND with an e-value of 1e-5 (Buchfink et al., 2015). Reference protein IDs of best hits were deployed to disentangle the taxonomic affiliation. The functional annotation was also performed for the non-redundant gene catalog. The non-redundant genes were aligned to Kyoto Encyclopedia of Genes and Genomes (KEGG) database (Ogata et al., 1999) and the Carbohydrate-Active enZymes (CAZy) database (Drula et al., 2022) using DIAMOND with an e value of 1e-5 (Buchfink et al., 2015).

### 2.6 Statistical analyses

All statistical analyses were performed using R software (R Core Team, 2021). Significant differences among groups were estimated by Duncan tests using R package “agricolae” (de Mendiburu, 2023). Pairwise correlation analysis between soil characteristics and composition of microbial community were calculated by mantel test using R package “ape” (Paradis and Schliep, 2019).

## 3 Results

### 3.1 Effects of crushed maize straw and corncob return on soil physicochemical properties

To evaluate the influence of distinct straw return materials on the soil quality, physicochemical properties were analyzed across four experimental treatments: CK (control), S (no straw return after 6 months), MS (crushed maize straw return after 6 months), CC (crushed corncob return after 6 months). Significant differences (*p* < 0.05) were detected among treatments for soil organic carbon (SOC), total nitrogen (TN), alkaline hydrolysis nitrogen (AN), available phosphorus (AP), and available potassium (AK) (Table 1). Compared to CK and S, treatments incorporating maize straw derivatives (MS and CC) exhibited elevated SOC, TN, AN, AP, and AK concentrations, indicating enhanced nutrient retention. Contrasting MS and CC treatments revealed divergent trends. CC demonstrated significantly higher SOC, TN, and AN content than MS, whereas AP and AK levels were reduced by 8%–14% under CC relative to MS. These findings underscore the material-specific effects of organic amendments on soil nutrient dynamics.

**TABLE 1:** Physical and chemical characteristics of soil under different maize straw return forms.

Properties	CK	S	MS	CC
SOC g kg^–1^	53.09 ± 1.88d	79.09 ± 3.57c	90.15 ± 5.87b	124.14 ± 8.68a
TN g kg^–1^	49.99 ± 4.12c	158.72 ± 6.08b	166.79 ± 11.78b	196.20 ± 12.16a
AN mg kg^–1^	34.92 ± 1.28d	85.13 ± 8.58c	124.49 ± 11.57b	145.49 ± 9.28a
AP mg kg^–1^	124.87 ± 3.79a	83.25 ± 4.84c	129.84 ± 4.81a	114.49 ± 19.95b
AK mg kg^–1^	79.67 ± 3.8d	243.15 ± 8.11c	328.01 ± 6.75a	301.08 ± 9.21b
pH	7.09 ± 0.08a	6.62 ± 0.09b	6.51 ± 0.03c	6.41 ± 0.05d

Values represent means ± standard deviation (*n* = 3). Different lowercase letters within the same row indicate significant differences among treatments at *p* < 0.05 according to Duncan’s test. SOC, soil organic carbon; TN, soil total nitrogen; AN, soil alkaline hydrolysis nitrogen; AP, soil available phosphorus; AK, soil available potassium.

Notably, treatment S (no straw return after 6 months) also showed marked increases in most physicochemical parameters compared to CK, with the exception of AP. This result could be attributed to seasonal temperature variability, particularly freeze-thaw cycles, which influence microbial activity and nutrient mineralization. Furthermore, soil pH in MS and CC treatments was marginally reduced compared to CK and S, suggesting organic matter decomposition and subsequent acidification processes.

### 3.2 Effects of crushed maize straw and corncob return on soil microbial diversity

Following the protocols described in the “Materials and methods” section, all samples generated more than 135 Mb of high-quality clean reads after quality control (Table 2). Assembly of these reads yielded contigs from 844,848 to 1,433,263 for each sample. N50 length ranged from 447 to 581 bp. Subsequent gene prediction generated a non-redundant gene catalog comprising 6,835,590 open reading frames (ORFs) for downstream analysis. Individual samples contained between 2,022,811 and 3,047,715 unique genes, reflecting substantial genetic diversity in all samples.

**TABLE 2:** Metagenomic sequencing and assembling statistics.

Samples	Clean reads	Clean base (bp)	Contigs	N50 (bp)	Unique gene number
CK_1	149,526,858	22,380,571,009	1,433,263	581	3,047,715
CK_2	142,655,340	21,339,368,866	1,312,189	567	2,914,767
CK_3	144,920,878	21,675,991,305	1,354,750	578	2,938,629
S_1	134,971,416	20,302,016,835	768,881	463	2,123,285
S_2	143,596,342	21,589,518,380	873,193	447	2,178,460
S_3	141,802,412	21,321,635,798	763,745	447	2,022,811
MS_1	143,854,194	21,628,621,883	971,310	464	2,484,425
MS_2	141,965,260	21,346,826,010	910,052	475	2,414,234
MS_3	135,072,508	20,317,075,523	844,848	459	2,187,743
CC_1	140,212,936	21,087,385,156	963,732	471	2,382,666
CC_2	140,314,680	21,105,461,338	1,108,049	541	2,595,663
CC_3	141,251,286	21,246,485,224	974,076	478	2,430,075

**FIGURE 1:**
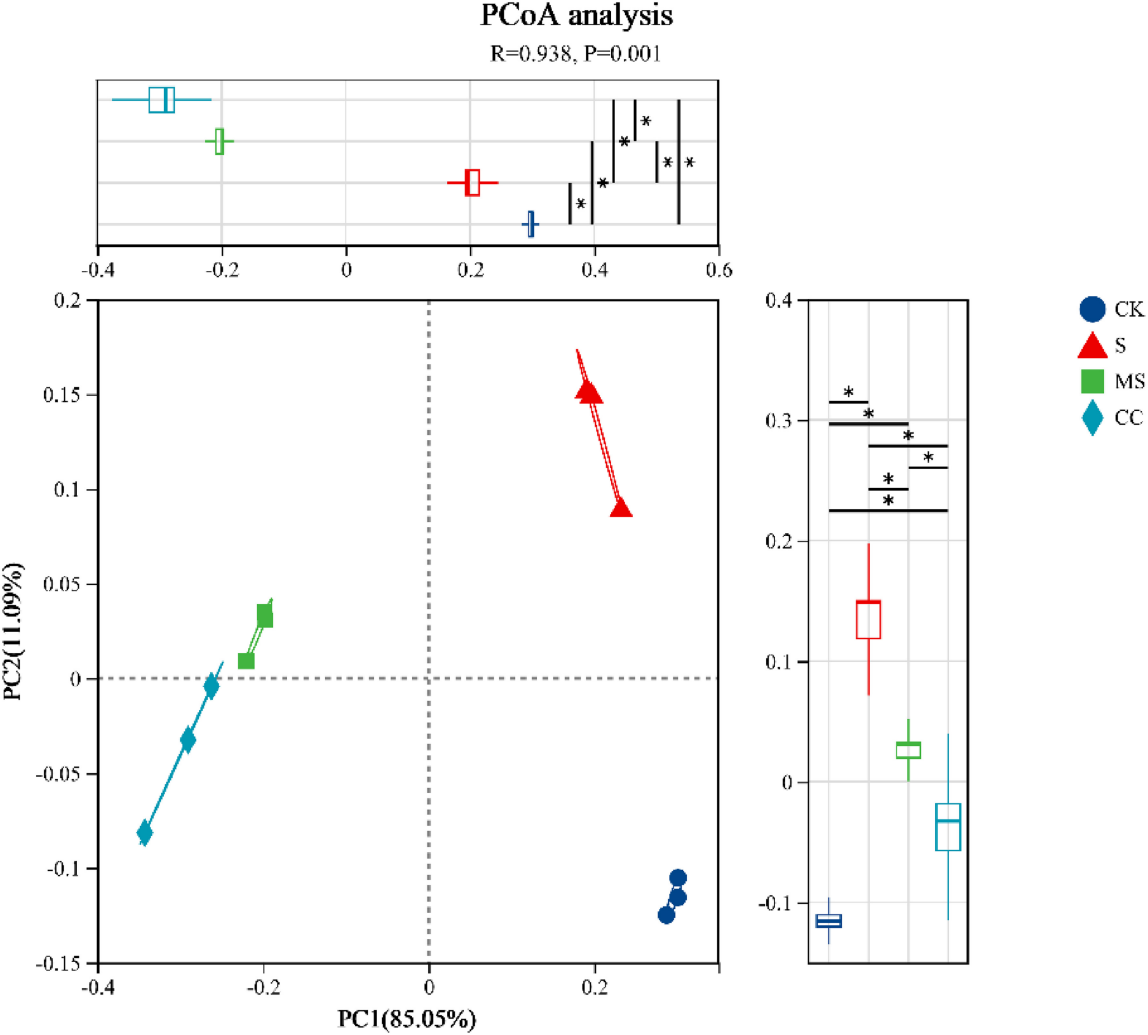
Principal coordinate analysis (PCoA) based Bray–Curtis distance of beta diversity. Comparing dissimilarity between groups was performed by ANOSIM test at PC1 and PC2. S indicates No straw returned over the same period; MS indicates crushed maize straw return; CC indicates crushed corncob return; CK indicates the experimental control. The detailed information was described in “Materials and methods.” *Indicates difference between any two groups.

The richness, diversity, evenness, and coverage of soil microbial communities were evaluated using the Chao, Shannon, Pielou_e, and Coverage indices of the soil microbial alpha diversity, respectively (Table 3). No significant differences (*p* > 0.05) were observed in richness, diversity, or evenness among the four treatment groups at the species level. However, coverage indices exceeded 99.9% in all groups, confirming sufficient sequencing depth to capture community diversity. These results suggest that maize straw return practices did not significantly alter microbial alpha diversity. In contrast, principal coordinate analysis (PCoA) based on Bray–Curtis distances revealed distinct clustering of microbial communities by treatment (Figure 1). The first two principal coordinates (PC1: 85.05%, PC2: 11.09%) collectively explained 96.14% of the total variance. Samples segregated into four clusters corresponding to treatments: communities from maize straw-amended soils (MS and CC) diverged markedly from non-amended controls (CK and S). Furthermore, microbial composition differed significantly (*p* < 0.01) between crushed maize straw (MS) and crushed corncob (CC) treatments, demonstrating that material type drives beta diversity shifts in soil microbial communities.

**TABLE 3:** Soil microbial alpha diversity analysis under different treatments.

Samples	Chao	Shanno	Pielou_e	Coverage
CK	12,420.67 ± 95.13a	6.08 ± 0.05a	0.65 ± 0.00a	1
S	11,640.33 ± 166.69b	5.99 ± 0.18a	0.64 ± 0.02a	1
MS	12,094.67 ± 211.28a	5.99 ± 0.02a	0.64 ± 0.00a	1
CC	12,172.33 ± 166.69a	5.87 ± 0.03a	0.62 ± 0.00a	1

Values represent means ± standard deviation (*n* = 3). Different lowercase letters within the same row indicate significant differences among treatments at *p* < 0.05 according to Duncan’s multiple range test.

**FIGURE 2:**
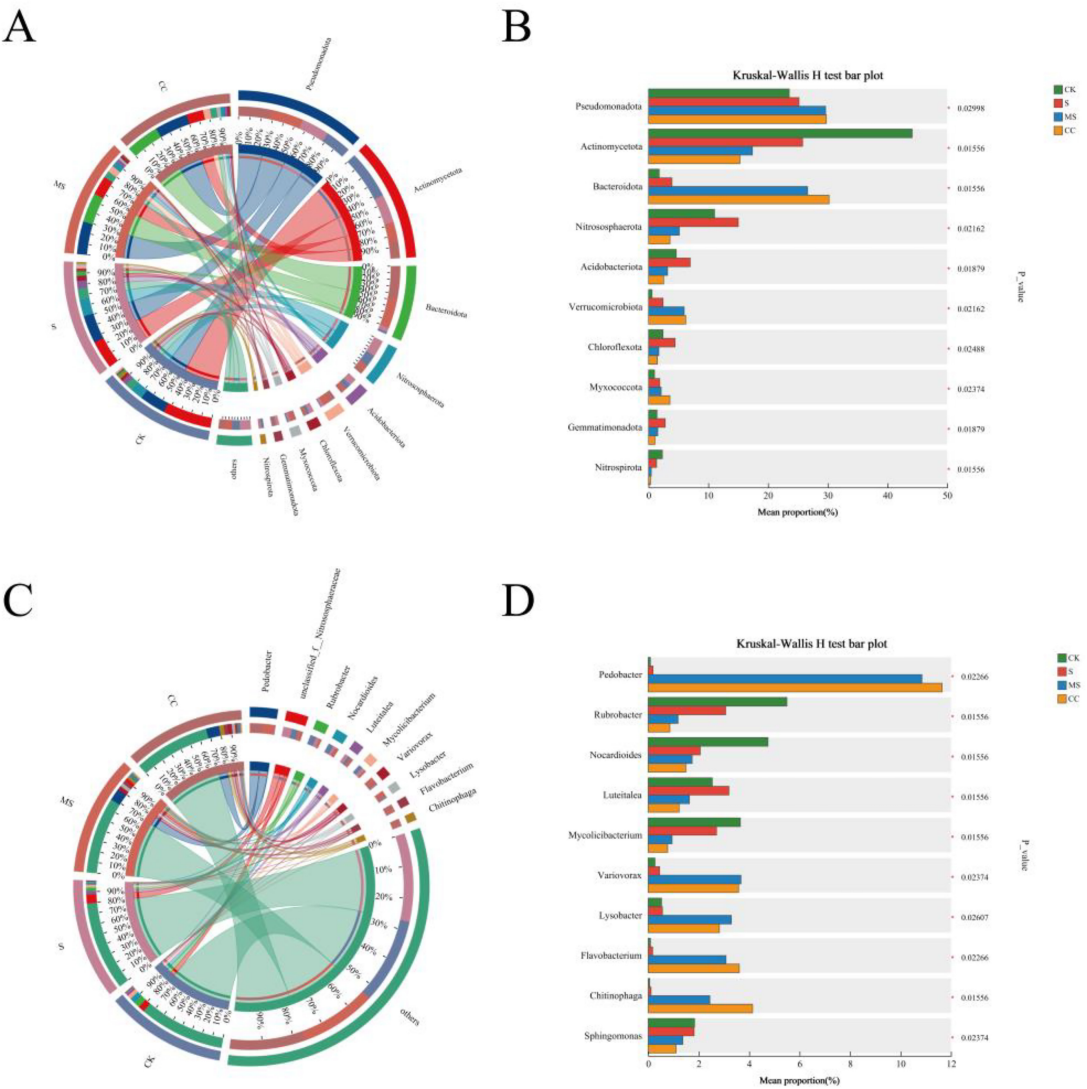
Microbial community composition under different straw returning treatments. **(A)** Relative abundance of the top 10 phyla across treatments. **(B)** Differential abundance analysis at the phylum level. **(C)** Relative abundance of the top 10 genera. **(D)** Differential abundance analysis at the genus level.

Soil microbial community abundance was analyzed at the phylum level (top 10 taxa) across four treatments: CK, S, MS, and CC (Figure 2A). Significant differences were observed between non-straw return treatments (CK, S) and straw return treatments (MS, CC) for all dominant phyla, with the exception of Pseudomonadota (Figures 2A, B). Actinomycetota dominated CK (44%) and S (26%) but declined markedly in MS (17%) and CC (15%). In contrast, Bacteroidota abundance increased substantially under straw return, comprising 27% (MS) and 30% (CC), compared to 2% (CK) and 4% (S). Nitrososphaerota exhibited a gradient decline across treatments and account for 11%, 15%, 5%, and 4% in CK, S, MS, and CC, respectively. Acidobacteriota abundance was highest in S (7%), followed by CK (5%), MS (3%), and CC (3%). Verrucomicrobiota abundance rose to 6% in MS and CC but remained minimal in CK (1%) and S (2%). Notably, Chloroflexota and Gemmatimonadota peaked in S, while Myxococcota abundance was highest in CC, exceeding levels in CK, S, and MS. Nitrospirota abundance was highest in CK (2%), declining to 1% in S and further decreasing to < 1% in MS and CC (Figure 2B).

Significant differences were observed in the relative abundance of the top 10 dominant genera across the four treatments (Figure 2C). Pedobacter was nearly undetected in non-straw return treatments (CK, S) but accounted for 11% (MS) and 12% (CC) of the community under straw return. Unclassified_f_Nitrososphaeraceae, the second most abundant genus, declined from 6% (CK) and 8% (S) to 3% (MS) and 2% (CC). Rubrobacter, the third most dominant genus, exhibited treatment-specific variations (Figure 2C). Straw return induced more pronounced shifts in genus-level composition compared to phylum-level trends. For instance, Pedobacter abundance increased dramatically in straw-amended treatments, with CC showing a 61-fold enrichment relative to S. Conversely, Rubrobacter and Nocardioides declined significantly in both MS and CC. Notably, Chitinophaga and unclassified_f_Nitrososphaeraceae abundances diverged sharply between straw return and non-amended treatments (Figures 2C, D). While Nocardioides decreased overall, transient increases were detected in specific straw return conditions, suggesting material-dependent microbial recruitment.

### 3.3 Effects of crushed maize straw and corncob return on soil microbial function

**FIGURE 3:**
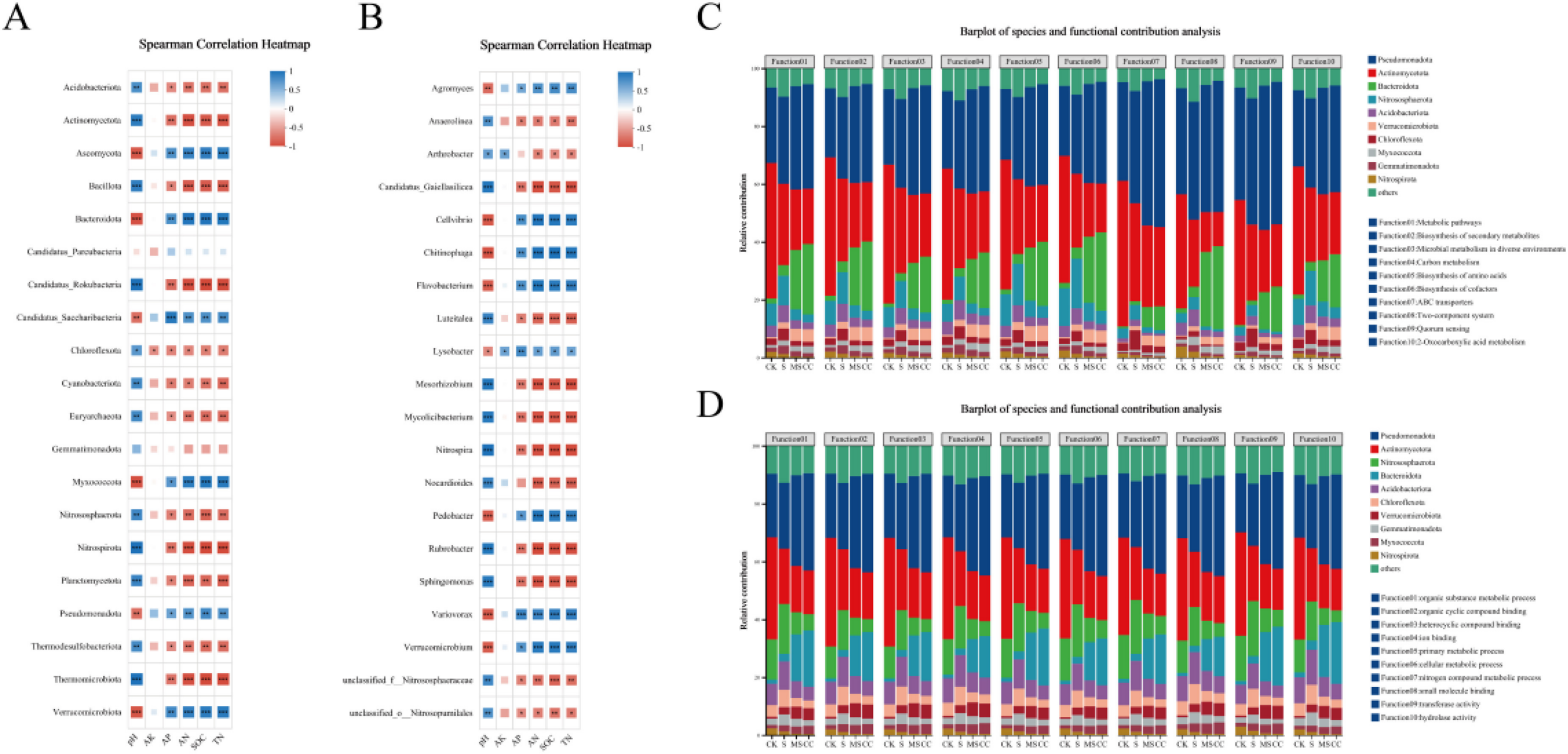
Correlations between soil physicochemical properties and the top 20 abundant phyla and genera and between species abundance and metabolic pathway contributions at phyla level and genera level. **(A)** Correlation between soil physicochemical properties and top 20 abundant phyla. **(B)** Correlation between soil physicochemical properties and top 20 abundant genera. **(C)** Correlation between species abundance and metabolic pathway (KEGG) contributions at phylum level. **(D)** Correlation between species abundance and GO function contributions at phylum level. SOC, soil organic carbon; TN, soil total nitrogen; AN, soil alkaline hydrolysis nitrogen; AP, soil available phosphorus; AK, soil available potassium. S indicates no straw returned over the same period; MS indicates crushed maize straw return; CC indicates crushed corncob return; CK indicates the experimental control. Spearman correlation was calculated. *represents *p* < 0.05, **represents *p* < 0.01, ***represents *p* < 0.001.

To assess the functional impacts of maize straw return on soil microbial communities, correlations between soil physicochemical properties and the top 20 abundant phyla/genera were analyzed. At the phylum level, available potassium (AK) exhibited no significant correlations with dominant phyla (Figure 3A). Soil pH showed a strong positive correlation with Bacillota, Actinomycetota, Candidatus_Rokubacteria, Nitrospirota, Thermomicrobiota, and Planctomycetota (*p* < 0.01), while these phyla were inversely correlated with soil organic carbon (SOC), total nitrogen (TN), and alkaline hydrolysis nitrogen (AN) (Figure 3A). In contrast, Ascomycota, Verrucomicrobiota, Bacteroidota, and Myxococcota demonstrated significant positive correlations with SOC, TN, and AN (*p* < 0.05) but were negatively associated with soil pH. Available phosphorus (AP) correlated positively only with Candidatus_Saccharibacteria (*p* < 0.01) (Figure 3A).

At the genus level, AK displayed no significant correlations with the top 20 genera except for weak associations with Lysobacter and Arthrobacter (Figure 3B). AP showed a highly significant positive correlation with Variovorax (*p* < 0.001). Soil pH and SOC/TN/AN exhibited opposing correlation pattern with dominant genera: taxa positively linked to pH were negatively associated with SOC, TN, and AN, and vice versa (Figure 3B). These results suggest that maize straw return indirectly modulates microbial functional profiles by altering soil physicochemical drivers such as pH and nutrient availability.

To elucidate linkages between microbial taxa and functional profiles, correlations between species abundance and metabolic pathway contributions were analyzed. The relative contributions of the top 10 phyla to 10 high-abundance KEGG pathways including Metabolic pathways, Biosynthesis of secondary metabolites, Microbial metabolism in diverse environments, Carbon metabolism, Biosynthesis of amino acids, Biosynthesis of cofactors, ABC transporters, Two-component system, Quorum sensing, and 2-Oxocarboxylic acid metabolism were quantified across treatments (Figure 3C). Pseudomonadota and Actinomycetota emerged as dominant contributors to these pathways in all groups, albeit with divergent trends. Pseudomonadota exhibited higher functional contributions in straw-amended treatments (MS, CC) compared to non-amended controls (CK, S), whereas Actinomycetota displayed an inverse pattern. Bacteroidota exhibited elevated functional contributions in MS and CC but played minimal roles in CK and S. Conversely, Nitrososphaerota and Nitrospirota were significantly enriched in CK and S relative to straw return treatments.

Similar analyses of Gene Ontology (GO) functions revealed strong contrasts between straw-returned (MS and CC) and non-straw returned (CK and S) groups, with distinct phylum-level contributions to organic substance metabolic process, organic cyclic compound binding, heterocyclic compound binding, ion binding, primary metabolic process, cellular metabolic process, nitrogen compound metabolic process, small molecule binding, transferase activity, and hydrolase activity (Figure 3D). These findings underscore how maize straw return reshapes microbial functional hierarchies, favoring taxa adapted to organic matter decomposition and nutrient cycling.

## 4 Discussion

### 4.1 Crushed maize straw and corncob return changes soil physicochemical properties

Straw return represents an effective management strategy for regulating soil nutrients and mitigating the losses of carbon (C), nitrogen (N), phosphorus (P), and potassium (K) in agricultural lands. Straw return is a process in which organic carbon is converted from crop carbon pools into soil carbon pools (Hao et al., 2019). Soil organic carbon is a vital indicator of soil fertility (Vilkienė et al., 2016). Given its high organic C content, crop straw is widely acknowledged as a valuable organic material for boosting soil organic C stocks, especially when supplemented with an appropriate amount of inorganic carbon (Beare et al., 2002). Our findings reveal that the incorporation of straw significantly improved the soil organic carbon (SOC), total nitrogen (TN), alkaline hydrolysis nitrogen (AN), soil available phosphorus (AP) and soil available potassium (AK) concentrations, whether crushed maize straw or crushed corncob was used. In addition, corncob return (CC) was more effective than maize straw return (MS) at increasing the content of soil organic carbon (SOC), total nitrogen (TN), and alkaline hydrolysis nitrogen (AN), while MS outperformed CC in enhancing soil available phosphorus (AP) and soil available potassium (AK) levels. We returned equivalent mass (straw or corncob) to fields individually. The previous study reported that maize straw contained 416.2 g/kg total carbon and 12.53 g/kg total nitrogen (Pei et al., 2015). In corncob, it contained 411.6 g/kg total carbon (Pahla et al., 2017). Therefore, these differences likely stem from variations in straw composition and decomposition dynamics, which influence nutrient release and stabilization mechanisms. The SOC pool is influenced not only by the degree of straw crushing but also by the specific components of the straw being incorporated. Koullas et al. (1992) reported that the decomposition rate of the straw accelerates with an increase in the degree of crushing, with a medium-speed decomposition rate of crushed straw limiting ineffective C loss and promoting organic C accumulation. Previous researches have also shown that the return of crop straw can enhance SOC stocks due to the relatively high C content of crop straw (Zhao et al., 2018; Cong et al., 2020). Furthermore, straw return is beneficial for C sequestration, primarily because of the increased humic acid C following straw incorporation (Hao et al., 2020). Fan et al. (2020) found that mixing soil with straw return markedly increased SOC content. They also observed enhanced macro-aggregate formation via straw incorporation further stabilizes SOC in surface soils. Additionally, the straw return treatment elevated SOC compared to the straw removal treatment (Yang et al., 2015), indicating that straw return to the field has significant potential for increasing SOC.

Straw return represents an effective strategy for mitigating nitrogen (N) and potassium (K) losses in agricultural soils, though its efficacy is contingent upon soil texture. The incorporation of straw into fields diminishes N loss by improving soil structure, which enhances water infiltration (Xia et al., 2018), and concurrently elevates soil N content. Furthermore, the increased soil organic carbon (SOC) resulting from straw return enhances cation exchange capacity (CEC), thereby reducing NH_4_^+^ leaching and improving the retention of NO_3_^–^ through the presence of deprotonated carboxyl groups. Research indicates that crop straw contributes significant amounts of K_2_O, a substantial proportion of P_2_O_5_, and a partial supply of N (Yin et al., 2018). Empirical evidence from Li et al. (2020) demonstrates that the combined application of straw return and potassium fertilizer elevates available K content by 72.9%, suggesting that straw mulching serves as an optimized K supplementation method for crops. As illustrated in Table 1, the influence of straw return on soil pH varies depending on straw feedstock, application rates, and soil type. However, the overall effect on soil pH remains marginal. Notably, a reduction in available phosphorus (AP) has been observed, likely attributable to the predominance of organically bound phosphorus in straw, which necessitates an extended mineralization period (Liu et al., 2024).

### 4.2 Crushed maize straw and corncob return shifts soil microbial community composition

Straw is a rich source of essential nutrient elements that promote soil microbial activity. The incorporation of different forms of maize straw into the soil significantly influenced microbial diversity, with observed shifts in specific microbial communities demonstrating a synergistic relationship with organic carbon dynamics (Figure 2). In this study, the application of maize straw—including both MS and CC treatments—did not markedly affect microbial alpha diversity (Table 2). However, principal coordinate analysis (PCoA) revealed distinct shifts in microbial beta diversity (Figure 1), a finding consistent with previous research by Liu et al. (2023). These results suggest that straw return alters the relative abundance of key soil microbial taxa, such as Actinomycetota, Bacteroidota, and Nitrospirae (Figure 2), thereby influencing overall microbial diversity.

Straw contains a range of nutrient elements that are beneficial to soil microorganisms. Various corn straw forms notably impacted soil microbial diversity through synergistic effects between microbial communities and organic carbon (Figure 2). In our research, the application of maize straw, including both MS and CC treatments, did not significantly alter microbial alpha diversity (Table 2), but drive beta diversity shifts in soil microbial communities according to the PCoA result (Figure 1). This result was aligned with the finding reported by Liu et al. (2023). This indicated that straw return can impact the relative abundance of some soil microbial taxa, such as Nitrososphaerota, bacteroidota, and Nitrospirota (Figure 2), and then have an effect on soil microbial diversity.

Soil as a dynamic reservoir of microbiota capable of decomposing diverse organic substrates. Consistent with established literature, our findings indicate that all experimental treatments were predominantly colonized by three bacterial phyla: Pseudomonadota, Actinomycetota, and Bacteroidota (Figure 2A), underscoring their key role in straw decomposition compared to other microbial taxa (Jurado et al., 2014; Su et al., 2020; Liu et al., 2024). The prevalence of Pseudomonadota suggests elevated carbon availability in the microenvironments of both MS and CC treatments, as this phylum is known to thrive in nutrient-rich soils (Liu et al., 2024). Meanwhile, Actinomycetota, renowned for their production of secondary metabolites, play a critical role in organic matter decomposition, particularly during later stages of straw degradation (Liu et al., 2024). Their widespread cellulase-synthesizing genes further facilitate cellulose breakdown, a pivotal step in straw decomposition (Du et al., 2022). Notably, Bacteroidota and Verrucomicrobia exhibited positive correlations with straw incorporation (Figure 2D), likely due to their metabolic specialization in recalcitrant carbon conversion, a key process in soil carbon and nitrogen cycling (Trivedi et al., 2015). In contrast, Actinomycetota displayed a negative association with straw return, possibly reflecting their oligotrophic adaptations and preference for low-carbon environments (Liu et al., 2024). The incorporation of maize straw into soil facilitates the release of nutrients and soluble organic matter, fostering a synergistic relationship with soil microbiota. This process enhances the proliferation of cellulolytic bacteria, thereby establishing a sustainable cycle that supports continuous organic matter decomposition and nutrient cycling.

### 4.3 Crushed maize straw and corncob return shifts soil microbial community functionality

The integration of maize straw into agricultural soils significantly influences microbial community structure and function, primarily through modifications to soil physicochemical properties. Our analysis revealed distinct correlations between dominant microbial taxa and key soil parameters, highlighting the central role of pH and nutrient availability in shaping microbial functional profiles. The strong positive association of Bacillota, Actinomycetota, and Planctomycetota with soil pH, coupled with their inverse relationship with SOC, TN, and AN, suggests niche differentiation between oligotrophic taxa adapted to higher pH conditions and copiotrophic groups thriving in organic-rich environments. This aligns with established ecological theory, where pH serves as a master regulator of microbial community composition (Fierer and Jackson, 2006). Conversely, the positive correlations of Ascomycota, Verrucomicrobiota, and Bacteroidota with SOC and nitrogen metrics underscore their role as key decomposers of straw-derived organic matter, consistent with their documented cellulolytic and proteolytic capacities (Trivedi et al., 2015).

The functional implications of these shifts were further elucidated through metabolic pathway analysis. The dominance of Pseudomonadota and Actinomycetota in KEGG pathways related to carbon metabolism, amino acid biosynthesis, and secondary metabolite production reflects their metabolic versatility in straw-amended soils. Notably, the enhanced functional contribution of Pseudomonadota under straw return conditions parallels their known ability to rapidly exploit labile carbon sources (Liu et al., 2024), while the decline in Actinomycetota activity may reflect competitive exclusion in high-carbon environments. The contrasting enrichment patterns of Nitrospirota and Nitrososphaerota in control soils (CK and S) further suggest that straw incorporation suppresses nitrifier populations, potentially redirecting nitrogen cycling toward immobilization pathways, a phenomenon with implications for N fertilizer management (Kuypers et al., 2018).

At the genus level, the minimal correlation between AK and microbial taxa (except for Lysobacter and Arthrobacter) contrasts with the strong linkage between AP and Variovorax, a genus renowned for phosphorus solubilization (Zheng et al., 2021). This dichotomy implies that straw-derived potassium may be primarily governed by abiotic processes, whereas phosphorus cycling is more tightly coupled to microbial mediation. The opposing correlations of taxa with pH versus SOC/TN/AN further reinforce the concept of resource partitioning, where copiotrophic genera (Bacteroidota) dominate high-organic matter microsites, while pH-tolerant taxa (Candidatus_Rokubacteria) occupy mineral-rich niches.

The GO term analysis provided additional resolution, revealing straw-induced shifts in microbial functional hierarchies. The heightened representation of organic compound metabolism, hydrolase activity, and nitrogen cycling pathways in MS and CC treatments aligns with the enzymatic demands of straw decomposition (Burns et al., 2013). Notably, the differential contributions of Bacteroidota (enriched in straw treatments) and Nitrospirota (depleted in straw treatments) to nitrogen metabolic processes suggest that straw return may favor assimilatory over dissimilatory nitrogen pathways—a potential mechanism for its observed N conservation effects (Xia et al., 2018).

In conclusion, maize straw return acts as an ecological selector, selecting for microbial consortia with enhanced organic matter processing capabilities while altering fundamental soil biogeochemical drivers. These findings provide a mechanistic basis for optimizing straw management protocols to harness microbial-mediated soil fertility improvements.

## 5 Conclusion

From harvest to the next planting season, corncob return improved key soil physicochemical properties more effectively than maize straw. Although microbial diversity was similar for both, the structure of the microbial community shifted in distinct ways. These shifts were associated with different dominant microbial groups. Importantly, analysis of microbial function showed that while both residues had an effect, the changes induced by corncob return were more substantial.

## Data Availability

The original contributions presented in the study are publicly available. All raw data can be found in NCBI under BioProject ID (PRJNA1348244).

## Footnotes

1
www.illumina.com

2
http://www.ncbi.nlm.nih.gov/Taxonomy/taxonomyhome.html/index. cgi?chapter=tgencodes
